# A Complex Case of Ornithine Transcarbamylase Deficiency in a Patient With Severe Comorbid Conditions

**DOI:** 10.1002/ccr3.72053

**Published:** 2026-02-19

**Authors:** Mobeen Khan, Sardar Ahmad Rafique, Suleman Khan, Asim Shah, Angraj Karan, Aizaz Anwar Khalid, Abdullah Afridi, Heela Tamim, Md Rubaiyat Tasfin Talukder

**Affiliations:** ^1^ Trainee Medical Officer, MTI Khyber Teaching Hospital Peshawar Pakistan; ^2^ Khyber Medical College Peshawar Pakistan; ^3^ Peshawar Medical College Peshawar Pakistan; ^4^ Department of Medicine Mymensingh Medical College Hospital Mymensingh Bangladesh

**Keywords:** hyperammonemia, inborn errors of metabolism, ornithine transcarbamylase deficiency, respiratory alkalosis, urea cycle disorder

## Abstract

Ornithine transcarbamylase deficiency (OTCD) is the most common urea‐cycle disorder, but it is often overlooked in Pakistan. It can present after the neonatal period with symptoms resembling sepsis and significant respiratory alkalosis. We report a 4‐month‐old boy with fever, lethargy, poor feeding, and loose stools for 5 days. He arrived with a rapid heart rate and quick breathing. His arterial blood gas showed a pH of 7.65, pCO_2_ of 14 mmHg, and HCO_3_^−^ of 15.5 mmol/L. Plasma ammonia levels were elevated at 78.1 μmol/L (reference range 11–32). A quantitative amino acid test revealed greatly increased glutamate (263 μmol/L; reference range 18–98) with low citrulline (6 μmol/L; reference range 16–32) and a normal ornithine/arginine ratio, which matches the pattern seen in partial OTCD. A contrast‐enhanced CT scan of the brain showed widespread leptomeningeal enhancement and mild enlargement of the bifronto‐temporal subarachnoid spaces. An echocardiogram found a small secundum atrial septal defect (ASD) and a tiny patent ductus arteriosus (PDA), with normal function in both ventricles. The patient was treated for suspected sepsis and high ammonia levels using sodium benzoate, L‐arginine, L‐carnitine, micronutrient support, and a low‐protein diet based on formula (Basic‐P/Morinaga BF‐1). This led to steady improvements in his clinical condition and blood work. This case highlights three key points for resource‐limited settings: (i) check ammonia levels early in any infant with unexplained encephalopathy and respiratory alkalosis; (ii) a low‐citrulline/normal‐ornithine profile should prompt evaluation for OTCD, even with mild hyperammonemia; and (iii) starting nitrogen‐scavenging therapy and adjusting dietary protein can prevent the need for dialysis. Documenting these presentations helps raise awareness in the region and emphasizes the importance of access to confirmatory molecular testing and family counseling when newborn screening is not available.

## Key Clinical Message

Early ammonia testing is critical in infants with unexplained encephalopathy and sepsis‐like features. Hyperammonemia with low citrulline suggests partial OTCD. Prompt nitrogen scavengers and protein restriction can stabilize patients and avoid dialysis. Early diagnosis enables timely genetic counseling.

## 1 Introduction

Ornithine transcarbamylase deficiency is a rare X‐linked disorder of the urea cycle, with an incidence of 1 in 50,000 to 80,000. It was first reported in 1962 by Russell in two girls, aged 20 months and 6 years, who were found to have hyperammonemia associated with episodic vomiting, delirium, stupor, failure to thrive, and mental retardation [1].

**FIGURE 1:**
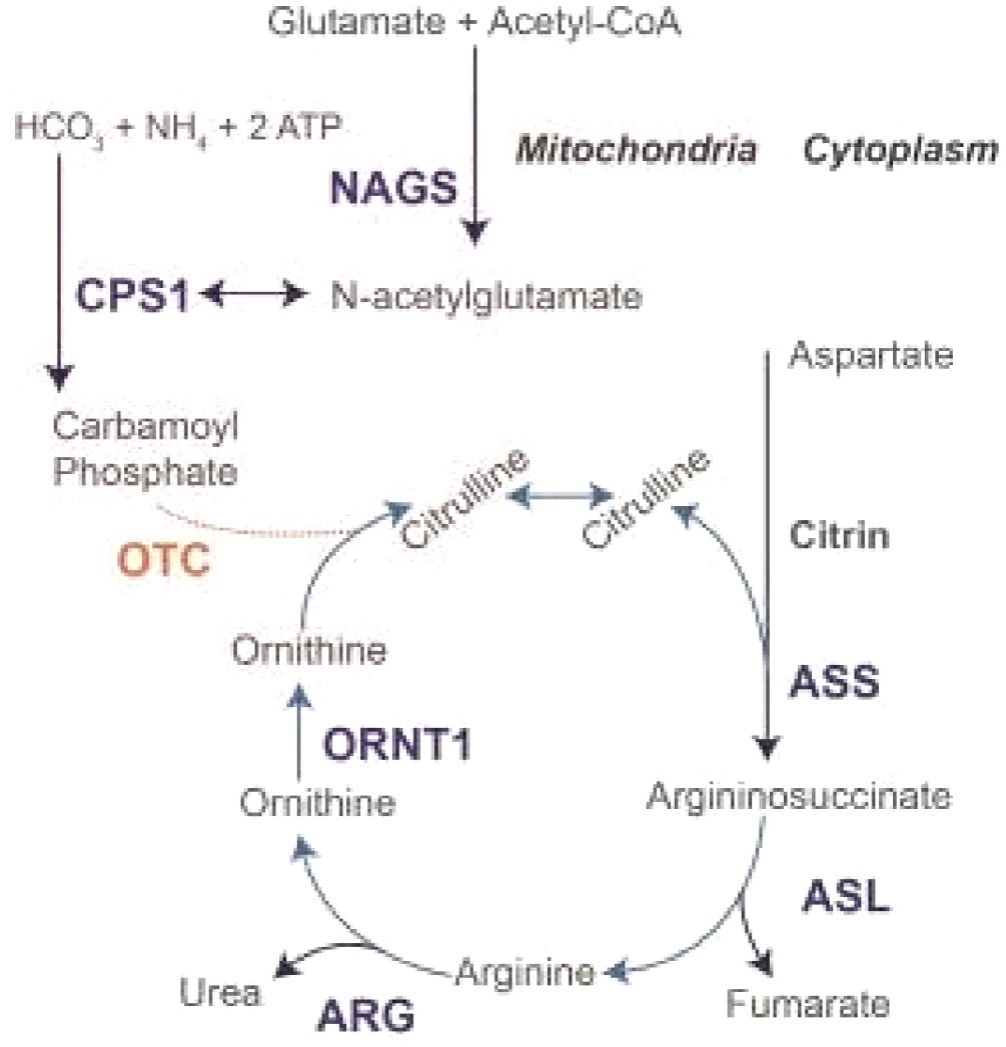
Urea cycle depicting the six enzymes involved and how OTC deficiency causes Ornithine buildup and decreases Citrulline production [2].

The urea cycle involves six enzymes that remove nitrogen from the body by converting neurotoxic ammonia into urea. One of these enzymes is Ornithine Transcarbamylase (OTC). A deficiency of this enzyme results in the excessive accumulation of ammonia, which is a potent neurotoxin, as shown in Figure 1 [2].

Ornithine transcarbamylase (OTC) deficiency can manifest as a severe neonatal onset disease in males and as a post‐neonatal onset disease (partial deficiency) in both males and females [2]. The infant becomes symptomatic after feeding starts because human milk supplies a protein load that is converted to ammonia. Severe neonatal onset Ornithine transcarbamylase deficiency typically appears on days two to three of life and is usually life‐threatening by the time medical attention is sought [3]. Symptomatic carriers often have a lifelong history of migraines, recurrent vomiting, abnormal behavior characterized by disorientation and confusion, anorexia, and avoidance of high‐protein foods [4].

Ornithine transcarbamylase deficiency (OTCD), although the most common urea cycle disorder worldwide, remains an exceptionally rare condition in Pakistan, with its true incidence unknown due to lack of newborn screening programs and underreporting; global estimates suggest a prevalence of 1 in 56,000–80,000 live births. In resource‐limited settings like Pakistan, delayed recognition and limited access to specialized metabolic diagnostics further increase morbidity and mortality. Therefore, documenting individual OTCD cases is crucial to raise clinical awareness, improve understanding of its presentation in the Pakistani population, and highlight the urgent need for early diagnostic strategies and multidisciplinary management to enhance patient outcomes.

## 2 Case History

A 4‐month‐old boy was born at 38 weeks via normal vaginal delivery to a mother with four prior pregnancies. He weighed 3.2 kg at birth. At 5 days old, he presented with fever, lethargy, poor oral intake, and loose stools. He had a delayed cry at birth and was admitted as a newborn for suspected sepsis. Upon arrival, he appeared pale and malnourished. He was tachycardic and tachypneic, with significant lethargy and poor feeding. His pulses were strong, the precordium was soft, and his heart sounds were normal.

### 2.1 Differential Diagnosis

Based on his presentation of lethargy, poor feeding, tachypnea, and a history suggestive of neonatal sepsis, the initial considerations included:

- Sepsis
- Inborn errors of metabolism (e.g., urea cycle disorders)
- Electrolyte disturbances
- Neurological causes (intracranial infection, structural abnormality)

### 2.2 Investigations

Serum urea was elevated at 77.4 mg/dL, while creatinine remained normal. Liver function tests were slightly abnormal during the acute illness but normalized before discharge. Arterial blood gas revealed a pH of 7.65, pCO_2_ of 14 mmHg, and HCO_3_^−^ of 15.5 mmol/L, indicating primary respiratory alkalosis with partial metabolic compensation. Plasma ammonia was 78.1 μmol/L (reference 11–32), indicating hyperammonemia.

A quantitative plasma amino acid profile showed elevated glutamate at 263 μmol/L (reference 18–98) and decreased citrulline at 6 μmol/L (reference 16–32). Arginine and ornithine were normal. This biochemical profile, together with hyperammonemia and respiratory alkalosis, strongly suggested ornithine transcarbamylase deficiency (OTCD). PTH‐intact was 32.66 pg/mL, within the normal range.

**FIGURE 2:**
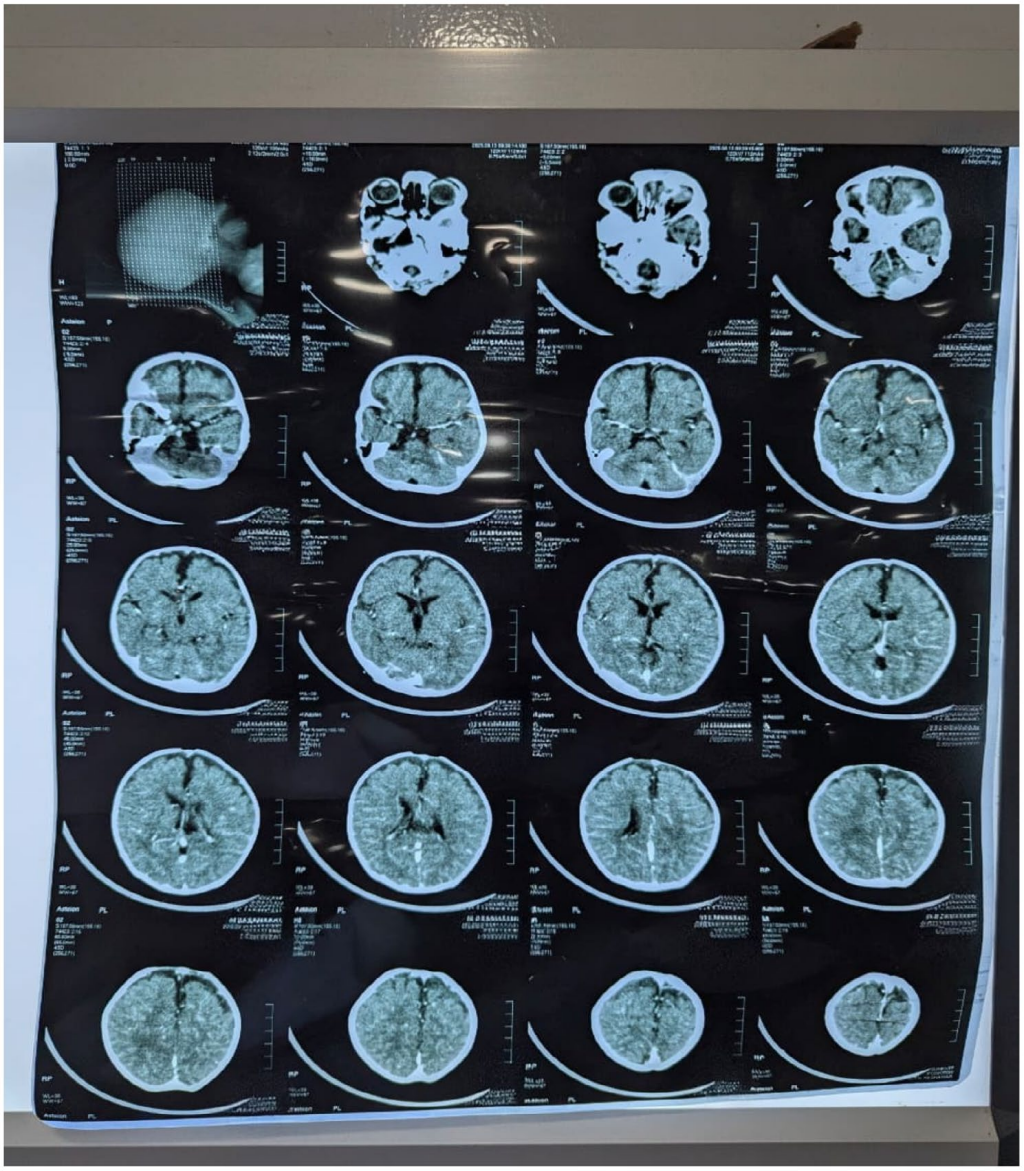
CT scan of the brain showing diffuse leptomeningeal enhancement and mild bifronto‐temporal subarachnoid space prominence (BESS pattern); no focal lesion or mass effect.

Contrast‐enhanced CT brain imaging revealed diffuse leptomeningeal enhancement with mild dilation of the bifronto‐temporal subarachnoid spaces (BESS pattern), with no focal lesions, mass effects, midline shifts, or hydrocephalus (Figure 2). Echocardiography showed a small ASD and a tiny PDA with left‐to‐right shunts, balanced chambers, good biventricular function, and normal cardiac situs. A single right SVC with a left innominate vein was noted. Outpatient cardiology follow‐up was advised in 6 months.

### 2.3 Treatment

The child was treated with broad‐spectrum antibiotics (meropenem and ceftriaxone/fortum) for suspected sepsis. Ammonia‐lowering therapy was initiated with sodium benzoate. Additional supportive treatments included L‐arginine, L‐carnitine, folic acid, zinc, and calcium. His feeding plan was adjusted to a protein‐restricted, formula‐based diet (Basic‐P/Morinaga BF‐1).

## 3 Conclusion and Results

The infant showed gradual clinical improvement with better feeding and a steady decline in ammonia levels. Liver tests normalized before discharge. Outpatient follow‐up was recommended, and the child was discharged in a stable condition.

## 4 Discussion

This case highlights several key points about ornithine transcarbamylase deficiency (OTCD). First, it demonstrates that partial or proximal urea‐cycle defects can present beyond the neonatal period with vague symptoms such as lethargy, poor feeding, and gastrointestinal upset, along with respiratory alkalosis that exceeds what the overall illness would suggest. These signs should trigger testing for ammonia levels, even if sepsis appears likely. The combination of high ammonia, low plasma citrulline, and normal levels of ornithine and arginine strongly indicates OTCD, which allowed for prompt treatment in this case. Including this reflex in pediatric emergency protocols is essential, especially in resource‐limited settings where delayed diagnosis can greatly worsen outcomes. International guidelines also emphasize that even mild hyperammonemia can damage the brain. This underscores the importance of rapid diagnosis and ammonia management, regardless of the peak value [5, 6, 7, 8, 9].

Second, the infant's imaging offers valuable teaching points. While CT suggests leptomeningeal enhancement and benign enlargement of the bifrontotemporal subarachnoid spaces (BESS), MRI with MR spectroscopy (MRS) is the most informative imaging method for urea cycle disorders. MRS can identify the characteristic increase in brain glutamine (along with decreased myo‐inositol), even if structural scans look normal. Changes seen on diffusion‐weighted imaging often involve the insular and peri‐insular cortex, the occipital lobes, the basal ganglia, and the dorsal thalamus during acute injury. These patterns are linked to neurocognitive issues in executive functions. When available, MRS helps distinguish metabolic encephalopathy from infectious meningitis when CT results are unclear [10, 11].

Third, management in this case follows established pathways. It involves quickly reducing ammonia levels by (i) temporarily stopping external protein intake and increasing calories to reverse catabolism, (ii) using nitrogen‐scavengers like sodium benzoate, phenylacetate, or oral phenylbutyrate, and (iii) administering urea‐cycle intermediates such as arginine. Data from an extensive multicenter study show improved survival with phenylacetate or benzoate during hyperammonemic crises. Dialysis is reserved for severe or refractory hyperammonemia, based on pediatric kidney replacement guidelines. The infant's relatively moderate peak ammonia allowed for a medical and dietary approach without dialysis, in line with existing algorithms [8, 9, 12, 13].

Fourth, having congenital heart disease (secundum ASD, tiny PDA) along with temporary kidney problems is a practical concern. Cardiorespiratory issues can mask the signs of respiratory alkalosis, and reduced kidney function can affect how the body clears benzoate and manages fluids during acute care. These concerns are specifically mentioned in guidelines that recommend close monitoring of heart and kidney function during therapy [5, 9, 13].

Ultimately, this case from a setting with limited screening underscores the importance of confirming the diagnosis through molecular testing and offering family counseling. Resources like *GeneReviews* emphasize that female relatives may be symptomatic carriers. Cascade testing, along with sick‐leave policies and emergency procedures, can help reduce the risk of future crises. Meanwhile, closely managing protein intake, reintroducing it once ammonia levels normalize, regularly monitoring amino acids, and having plans for early escalation are essential for safety in outpatient care [6, 7, 8, 9, 14].

## Author Contributions

**Mobeen Khan:** conceptualization, methodology, project administration, supervision. **Sardar Ahmad Rafique:** visualization, writing – original draft, writing – review and editing. **Suleman Khan:** conceptualization, methodology, writing – original draft, writing – review and editing. **Asim Shah:** validation, visualization, writing – original draft. **Angraj Karan:** writing – original draft, writing – review and editing. **Aizaz Anwar Khalid:** methodology, resources. **Abdullah Afridi:** methodology. **Heela Tamim:** methodology, writing – original draft. **Md Rubaiyat Tasfin Talukder:** visualization, writing – review and editing.

## Funding

The authors have nothing to report.

## Ethics Statement

The authors have nothing to report.

## Consent

Written informed consent was obtained from the patient and/or their legal guardian for the publication of this case report, including relevant clinical details and any accompanying images.

## Conflicts of Interest

The authors declare no conflicts of interest.

## Data Availability

The data used in this case report were obtained from a patient who presented to our hospital. All referenced studies and background information are publicly available on databases such as PubMed and Google Scholar. No additional datasets were generated or analyzed for this study.
